# Evaluating Cardiovascular Patient Support Groups: A Cross-Sectional Control-Group Questionnaire Study of Patients and Healthcare Providers

**DOI:** 10.3390/healthcare13212692

**Published:** 2025-10-24

**Authors:** Dana Stefanovic, Julia Pantoglou, Lisa Voggenberger, Fabian Bekelaer, Markus Mader, Erika Zelko

**Affiliations:** Institute of General Practice, Johannes Kepler University Linz, 4040 Linz, Austria; dana.stefanovic@sydney.edu.au (D.S.); lisa.niehoff@jku.at (L.V.); fabian.bekelaer@jku.at (F.B.); markus.mader1@gmx.at (M.M.); erika.zelko@jku.at (E.Z.)

**Keywords:** cardiovascular diseases, patient support group, self-help groups, cardiac rehabilitation, long-term cardiac rehabilitation, community-based rehabilitation, health literacy, social support, self-management

## Abstract

**Background/Objectives**: Participation in patient support groups (PSGs) for long-term cardiac rehabilitation (CR) enables sustainable, cardioprotective lifestyle modification, which can reduce morbidity and cardiovascular mortality. This study evaluates a nationwide network of cardiovascular PSGs in Austria in the holistic management of patients with cardiovascular disease (CVD). **Methods**: Between December 2023 and March 2024, we evaluated PSGs by surveying members about their knowledge of CVD and self-assessed health status, comparing them with non-member CVD patients. Members’ and healthcare providers’ perceptions of the program were also assessed. **Results**: PSG members rated their own health literacy regarding CVD higher than non-members (median score: 7.00 vs. 6.00, range 1–10; U = 7088.00, *p* = 0.014). These results were not confirmed by an objective assessment of participant knowledge, and members did not exhibit better health data. However, members appreciated the association’s services in providing an important social network, enabling them to feel empowered in managing their condition (52.2%), and stated that it “improved their quality of life” (69.6%), while rating detrimental effects of participation as low. Healthcare providers also viewed the program rather positively, though only 5% reported prior contact with it. **Conclusions**: Cardiovascular PSGs offering long-term CR provide valuable support and are appreciated as important social networks. While further research is needed to confirm improvements in health literacy or health status, participation addresses patients’ psychosocial needs and may particularly benefit those at risk of social isolation and depression, therefore offering a useful addition to comprehensive CVD care.

## 1. Introduction

Chronic non-communicable diseases, particularly arterial hypertension and diabetes, are a growing public health issue, exacerbated by demographic changes, unhealthy lifestyles, pollution, and urbanization [1]. In Austria, 27% of inpatients have cardiovascular diseases (CVDs), the focus of this study, with 38% of deaths attributed to them [2]. Treatment of these conditions is complex, and poor adherence leads to recurrent CV events and increased healthcare costs [3].

To address these challenges, primary care models aim to provide holistic, multidisciplinary care, focusing on community engagement and patient self-management [4]. However, implementation is hindered by issues like isolated healthcare settings, patient motivation difficulties, and clinical information system errors, creating a gap between theory and practice [5].

Limited health literacy, found in up to 60% of cardiac patients [6,7], is linked to poorer health behaviors, higher hospital readmission rates, and reduced quality of life [8,9]. Interventions to improve health literacy can enhance social support and patient interaction with healthcare systems [10].

Cardiac rehabilitation (CR) has been shown to improve health literacy [11] and reduce the risk of myocardial infarction, mortality, hospitalizations, and healthcare costs, while improving health-related quality of life (HRQoL) for patients with coronary heart disease [12]. Core CR components include nutrition counseling, risk factor modification, psychosocial management, education, and exercise [13], and a comprehensive care model is recommended [14].

Internationally, different systems are used to categorize the various phases of CR, from acute inpatient CR to long-term outpatient CR. Outpatient CR can be conducted in an organized community setting like a patient support group (PSG). While the effectiveness of peer support is not clear [15], there is evidence that PSGs have the potential to increase health literacy [16] and to have a positive effect on depression, anxiety, quality of life, self-care, and medication adherence [17].

In Austria, the Austrian Heart Association (AHA, Österreichischer Herzverband) plays an important role in managing long-term (phase IV) CR. It consists of a nationwide network of PSGs and offers a program of exercise-based as well as psychosocial and educational activities centered around regular meetings in a community setting. Although the program has been shown to have an impact on engagement in health-promoting sports, stress reduction, and dietary adjustments [18], to our knowledge, its impact on health literacy, morbidity, patient satisfaction, and perceptions has not been assessed. This study aims to evaluate the Austrian PSG program more comprehensively and to assess its effectiveness in supporting patients with CVD.

## 2. Materials and Methods

### 2.1. Study Design and Objectives

This cross-sectional study involved participants that completed a customized questionnaire based on their group (PSG members, non-members, or healthcare providers). The primary goal of this study was to evaluate whether CVD patients in the PSG network demonstrate better disease knowledge than non-members, based on the assumption that regular information and personal exchange through the AHA enhance understanding. Additionally, the influence of participation on personal health data and HRQoL and the perceptions of PSG members and healthcare providers regarding the AHA and its program were assessed.

### 2.2. Participants and Criteria for Inclusion

Participants were divided into three groups:

1. PSG members (individuals in the AHA);
2. Control group (CVD patients not in the AHA);
3. Healthcare providers (unrelated professionals in targeted fields).

Participants in the member and control patient groups were included if they reported a diagnosis of at least one cardiovascular disease (CVD). To be included in the member group, participants had to report being members of the PSG association, while control-group patients reported no affiliation. Datasets from healthcare providers were included if respondents indicated that they belonged to one of the targeted health professions. Incomplete datasets that did not contain any relevant data were defined as not meeting the criteria for inclusion.

### 2.3. Recruitment and Data Collection

Data was collected between December 2023 and March 2024. PSG members were invited through local group meetings, a members’ magazine, and a national meeting of the PSG association. Regional AHA group leaders who facilitated data collection from members received training on study procedures and bias prevention, organized by the Institute of General Medicine, Johannes Kepler University Linz.

Control-group patients and healthcare providers were recruited from general practitioner (GP) offices, hospitals, and universities, via a quality circle for internal medicine/cardiology, and from a medical laboratory via invitations, direct contact, and snowballing. The control group was independently recruited and not matched to PSG members on all characteristics.

The intended sample size was based on the estimated number of active Austrian Heart Association members at the time of the study, serving as the reference population for one of the target groups. Recruitment was conducted with the aim of obtaining comparable group sizes across the three populations to allow meaningful between-group comparisons. However, as indicated by the differing response rates, the final sample size of 548 participants reflected realistic participation rather than a predefined power calculation.

As participants did not receive individual invitations, the exact number of individuals reached and response rates could not be determined. To aid interpretation of the sample composition, the size of each target population was roughly estimated as follows: approximately 1400 PSG members, around 1500 patients with cardiovascular disease (non-members), and 600–1000 healthcare providers.

### 2.4. Instruments

A customized questionnaire was developed based on a conceptual framework aligned with the research questions. It underwent pilot testing and cognitive debriefing [19] to ensure clarity and relevance.

The questionnaire for PSG members and the control group had five sections:

- Demographics: age, gender, marital status, nationality, and education level.
- Health literacy: Multiple-choice questions on CVD knowledge and a self-rated score of CVD knowledge (range 1 to 10). This section was developed by the authors drawing on their clinical and academic expertise in cardiovascular health. Items were refined through cognitive debriefing within a multidisciplinary team of healthcare experts and pilot-tested within a small sample of participants (*n* = 15) representing all three target groups, to ensure clarity, comprehensibility and content validity. The instrument has not yet undergone formal psychometric validation and should therefore be regarded as a pragmatic, context-specific tool.
- Personal health status: Self-reported diagnoses, recent doctor visits, medications, blood pressure measurements, weight, height, and blood sugar and blood lipid levels.
- HRQoL: assessment via the EuroQoL EQ-5D-5L [20].
- Perception of the PSG program (PSG members only): This section was intended to be exploratory in nature and aimed to provide a preliminary understanding of the perceived benefits of PSG participation. The Theoretical Framework of Acceptability (TFA) tool [21], which contains seven constructs (affective attitude, burden, perceived effectiveness, ethicality, intervention coherence, opportunity costs, self-efficacy), was used after forward and backward translation into German and validated via cognitive debriefing, and with permission of the authors [19].

Healthcare providers completed a separate questionnaire on their interactions with PSGs, their perceptions of patient and physician benefits, and the importance of doctor–PSG collaboration. The TFA tool was also used to assess their perception of the AHA’s work.

### 2.5. Data Analysis

Data were analyzed using IBM SPSS Statistics (IBM Corporation, Armonk, NY, USA, Version 29) and Microsoft Excel (Microsoft Corporation, Redmond, WA, USA, Version 16.0). Descriptive statistics summarized the demographic and clinical characteristics of participants. Due to mismatched sample sizes and age distribution differences between groups, the non-parametric Mann–Whitney U test was used for continuous variables, while χ^2^ tests were employed for categorical variables. Fisher’s Exact Test and Monte Carlo simulation *p*-values were applied when expected cell counts were below 5. Subgroup analyses explored variations within participant subsets where appropriate. Statistical significance for the main hypothesis was set at *p* < 0.05. *p*-values for all other analyses conducted to contextualize findings were considered potential trends for further investigation, rather than evidence of significant differences. EQ-5D-5L index values were calculated using the German value set [22]. A sensitivity analysis was conducted excluding medical students from the healthcare providers analysis to assess whether their inclusion affected key outcomes.

### 2.6. Ethical Considerations

This study was approved by the Human Research Ethics Committee of the Johannes Kepler University Linz, Austria (approval numbers 1091/2023, 1093/2024). Informed consent was obtained from all participants.

## 3. Results

A total of 257 PSG members, 79 non-members, and 270 healthcare providers participated. After excluding 58 incomplete datasets (6 for PSGs, 3 for controls, 49 for healthcare providers), 548 datasets were analyzed.

PSG members and controls differed significantly in age, gender, marital status, education, and disease duration. Members were older, more likely female, and less often married, had lower educational levels, and had longer cardiovascular disease histories (see Table 1 for details).

Healthcare providers included GPs (33.9%), nurses (22.6%), medical students (12.2%), physiotherapists (8.1%), internal medicine specialists (7.7%), and occupational therapists (1.4%), among others (13.6%) (See also Appendix A.1, Table A1).

### 3.1. Health Literacy

PSG members rated their CVD knowledge significantly higher than non-members (median score: 7.00 vs. 6.00, range 1–10; U = 7088.00, *p* = 0.014) (see Table 2).

Members also felt better informed about their illness (93.2% vs. 73.4% chose “well informed” or “very well informed”, χ^2^(3) = 23.339, *p* < 0.001) (see Appendix A.2, Table A2).

Regarding objective assessment of CVD knowledge, significant differences were found in specific questions about CVD risk factors and symptoms, but overall, there was no significant difference between the groups (see Appendix A.2, Table A3).

The PSG-member group considered the AHA (76.9%) the most important source of information about their own CVD and healthy lifestyle, followed by specialists (72.9%) and GPs (53.8%). The control group rated specialists (63.2%), GPs (52.6%), and the internet (32.9%) as the most important sources (See Table 3).

### 3.2. Self-Reported Health Data

**Figure 1:**
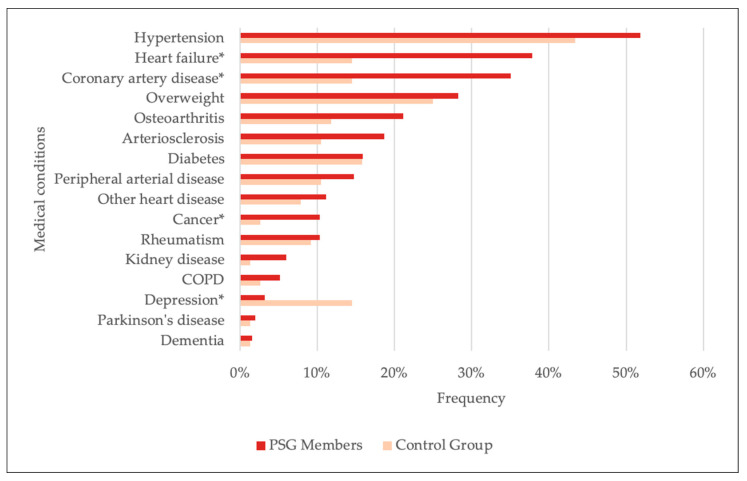
Prevalence of medical conditions reported by PSG members and control-group patients. Significant differences between groups are indicated, highlighting key variations in health status (* = significant difference; COPD = chronic obstructive pulmonary disease).

The most common conditions among PSG members and control patients are displayed in Figure 1.

Significant differences between groups were observed for the following conditions: heart failure (χ^2^(1) = 12.399, *p* < 0.001), coronary artery disease (χ^2^(1) = 9.834, *p* = 0.002), and cancer (χ^2^(1) = 3.926, *p* = 0.048) were more common in PSG members, while depression was more common in the control group (Fisher’s Exact Test, *p* < 0.001) (See also Appendix A.3, Table A4 for details).

Most PSG members suffered From 3 To 6 Conditions (49.0%) or 0 to 2 conditions (48.2%). In contrast, the control group had more participants with 0–2 conditions (68.6%) and fewer with 3–6 (30.0%). The higher multimorbidity burden in PSG members was potentially significant (Fisher’s Exact Test, *p* = 0.015) (See also Appendix A.3, Table A5 for details).

Similarly, members also indicated suffering from their CVD for longer (median: 12 vs. 5 years; U = 2264.00, *p* < 0.001) (See also Appendix A.3, Table A6 for details).

Regarding key indicators of cardiovascular risk (including BMI, blood pressure, blood sugar levels, and lipid profiles), no statistically significant group differences were observed for BMI, blood sugar, or lipid profiles A statistically significant difference did emerge in self-reported blood pressure categories (χ^2^(3) = 8.463, *p* = 0.037), with a higher proportion of control participants classified as having Grade II hypertension. Despite this, overall cardiovascular risk profiles appeared largely comparable between PSG members and control-group participants (See Table 4 for details).

PSG members reported regularly taking a median of 3.0 medications, while the control group reported taking only 2.0 (U = 2663.50, *p* = 0.008) (See Appendix A.3, Table A7).

Members were generally more likely to provide personal health data than control group-participants, with a statistically significant difference in BMI reporting (92.0% vs. 84.2%, χ^2^(1) = 4.042, *p* = 0.044). No significant differences were observed in the remaining parameters, including blood pressure and blood sugar and lipid levels (See Appendix A.3, Table A8).

### 3.3. Health-Related Quality of Life (HRQoL)

The median EQ-5D-5L utility (index) score was slightly higher for PSG members (0.943, IQR = 0.120 vs. 0.913, IQR = 0.173; U = 6562.00, *p* = 0.251) (See Appendix A.4, Table A9). While the overall index did not differ significantly between groups, a significant difference emerged at the domain level, with PSG members reporting less depression and anxiety (χ^2^(3) = 18.077, *p* < 0.001) (See Appendix A.4, Table A10 for details).

Similar findings with a slightly higher median value of 80.00 (IQR = 25) among PSG members vs. 75.00 (IQR = 38) in the control group were also noted for self-rated current health status (range of 0–100; U = 6982.50, *p* = 0.484) (See Appendix A.4, Table A11).

### 3.4. Perception of the Program

#### 3.4.1. PSG Members

**Figure 2:**
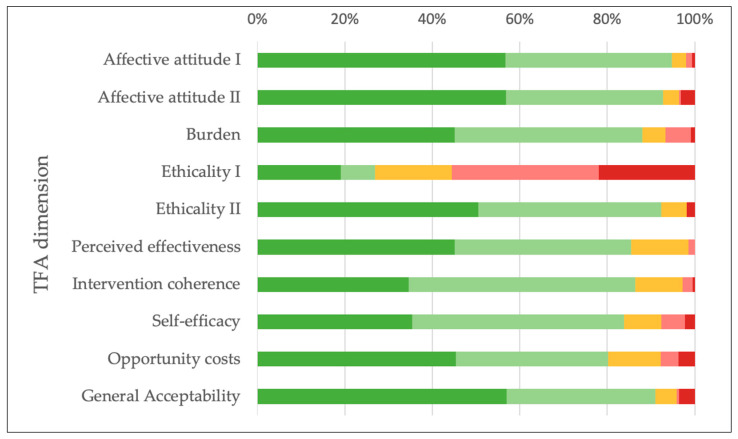
Percentage distribution of PSG members’ responses within each TFA domain, based on those provided a response. Response options are displayed using a color gradient ranging from dark green (most beneficial) to dark red (most detrimental) to indicate the perceived effects of PSG participation. Detailed counts and response categories are presented in Appendix A.5 in Table A12.

Members displayed a positive overall attitude toward the program of the association, as assessed via the TFA questionnaire. The vast majority of respondents indicated that they (strongly) liked the program (94.8%), felt (very) comfortable participating in its activities (92.7%), and found it (completely) acceptable (91.0%). Most (85.4%) agreed (strongly) that it had strengthened their ability to manage their CVD, 86.4% indicated that it was (very) clear to them how it would help them manage their CVD, and 83.9% felt (very) confident in engaging with the program. While for only 26.8% of members, moral or ethical issues were not (at all) considered to be associated with participation, 92.4% considered the program to be fair or very fair. Burden and opportunity costs were also rated as low, with 88.1% stating that participating in the activities cost them no effort at all or little effort, and 80.3% indicating that engaging with the program did not interfere (at all) with their other priorities Percentages represent combined totals of the two positive response categories regarding PSG participation (See Figure 2 and Appendix A.5, Table A12 for details).

When asked about the personal benefit of participating in the program, the statement that members, in particular those who were widowed (79.2%), most frequently agreed with was that participation “improved [their] quality of life” (69.6%). The majority of the participants (52.2%) also agreed that “the Heart Association constitutes an important social network” and “strengthens [them] in [their] ability to deal with [their] own illness”. About half (48.2%) considered the PSG network an “important source of trustworthy medical information”. For 32.4% of respondents, the association helps them navigate the healthcare system, and for 22.7%, it “provides [them] with support [they] do not receive from any doctor or other healthcare professional” (See Appendix A.5, Table A13).

#### 3.4.2. Healthcare Providers

**Figure 3:**
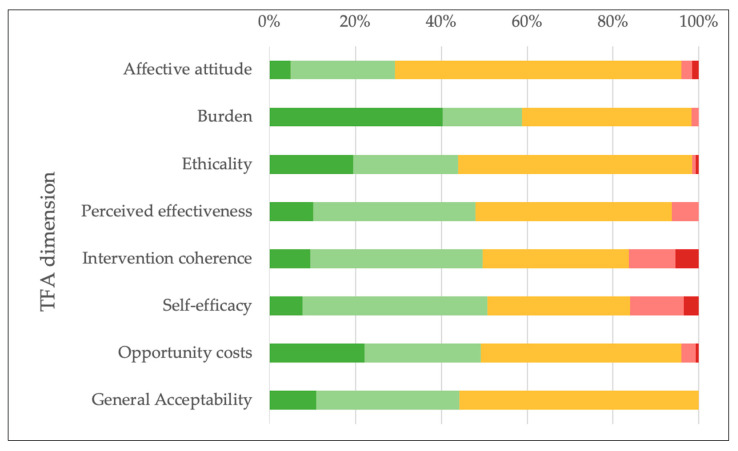
Percentage distribution of healthcare providers’ responses within each TFA domain, based on those provided a response. Response options are displayed using a color gradient ranging from dark green (most beneficial) to dark red (most detrimental) to indicate the perceived effects of PSG participation. Detailed counts and response categories are presented in Appendix A.5 in Table A14.

Of the healthcare providers, 51.6% completed the TFA questionnaire fully. Five percent indicated ever having had contact with the AHA. However, overall, they displayed a slightly positive attitude toward its activities, with 29.3% indicating that they (strongly) liked the program and 44.1% finding the program (completely) acceptable. About half were (very) sure that those affected would be able to partake successfully (50.7%), (strongly) agreed that the AHA had strengthened the ability of its members to manage their CVD (48.0%), or were (very) clear on how it would help them do so (49.7%). Many (43.9%) (strongly) disagreed that there are negative moral or ethical consequences associated with participation. The majority (58.8%) stated that participating in the activities had cost them no effort at all or little effort, and 49.1% that engaging with the program had not interfered (at all) with their other priorities (Percentages represent the combined totals of the two positive response categories regarding PSG participation). The option “no opinion” was chosen frequently across all dimensions (46.5% of responses) (See Figure 3 and Appendix A.5, Table A14 for details).

The sensitivity analysis excluding medical students (*n* = 27) from the analysis resulted in small changes in healthcare providers’ perceptions of the AHA. The largest change was a +2.9% increase in those who “strongly disagreed” that AHA collaboration interferes with their priorities (Opportunity Costs). Confidence in patients’ ability to participate rose by +1.0%, and “strong agreement” that the AHA helps patients increased by +1.4%. Neutral responses declined slightly in some domains, but this was not a consistent trend across all domains. Overall, the changes were minor, but showed slightly more favorable and decisive response patterns compared to when medical students were included in the analysis (See Appendix B, Table A15 for details).

## 4. Discussion

The main results of the present study are as follows: Objective CVD knowledge did not differ significantly between PSG members and non-members. However, self-perceived knowledge was higher among members. PSG members had more health conditions and a longer CVD duration, yet they did not display significantly worse health data, including HRQoL. Notably, depression was more common in the control group. Members and healthcare providers displayed a positive attitude toward the association’s program, but only 5% of the latter group had previous contact with it.

While previous studies suggest that CR and PSG participation can improve health literacy [11,16], our study did not confirm this in terms of objective CVD knowledge, with both PSG members and non-members showing knowledge gaps. For example, only half of the PSG members identified diabetes or poor diet as CVD risk factors, though they were more informed about anxiety/fear of dying as a sign of a heart attack. Despite lacking objectively better knowledge, PSG members felt better informed and were more likely to report their health data. While this could be due to other factors (e.g., willingness to share sensitive information, age, cognitive function, and level of education), it may reflect either a better understanding of one’s health or a greater appreciation of its importance.

A notable finding of our study was that members appeared less likely to suffer from depression. Although we could not establish a causal relationship, previous research has suggested that peer support may help alleviate depression and anxiety [23]. However, this finding should be interpreted with caution, as depression is influenced by factors such as age, comorbidities, and disease duration. While PSG members had more comorbid conditions and a longer CVD duration than the control group, they were also older. This age disparity thus likely affected certain health outcomes, particularly those sensitive to age-related factors. It is therefore possible that the observed differences reflect both peer support effects and confounding. Although our sample size did not allow for age adjustment, future research with larger, age-matched cohorts or statistically controlled analyses could further clarify its influence, thus allowing for more precise comparisons. Despite these findings, the self-reported health data of PSG members were not found to be significantly worse, with these participants even showing a trend toward better BMI and reporting better blood pressure values.

Previous research indicates that patients with poorer reported health status at baseline are most likely to sign up for peer support self-management interventions, but participants who engage in such activities are more likely to have better baseline health status [24]. Additional information on the members’ health status before they joined a PSG would have provided insight when comparing them to the control group.

Even though there is evidence that peer support interventions can improve HRQoL and alleviate psychological complications in cardiac patients [17]. This could not be confirmed in our study, as the HRQoL values did not appear to differ significantly between members and non-members. However, the statement that members agreed with the most when asked about the perceived benefit of participating in PSG activities was that it “improves [their] quality of life”. To establish a causal relationship, it would have once again been helpful to know the members’ baseline HRQoL ratings prior to them joining a PSG.

Due to the limitations of our study in terms of its design and relatively small sample size, it was not possible to control for potential confounders or perform meaningful subgroup analyses. Future research using age- and education-matched cohorts, longitudinal tracking of participants, randomization where feasible, and more-objective outcome assessments (e.g., clinically obtained health parameters, functional capacity tests, or data on rehospitalizations) would allow for more-precise impact assessments.

As expected, PSG members rated the Austrian Heart Association favorably when asked about its acceptability and the benefits of membership. The majority of members perceived participation in the program as beneficial for their quality of life and as offering important social benefits and a sense of empowerment. While these perceptions are consistent with previous research showing that peer support can improve self-efficacy [25] and psychosocial outcomes [26], our findings are based on self-reported attitudes rather than objective measures and should therefore be interpreted with appropriate caution. Interestingly, 23% of members stated that “The Heart Association provides [them] with support [they] do not receive from any doctor or other healthcare provider”.

Our surveyed healthcare providers also agreed with the potential benefits of PSGs to some degree, even though many chose to respond with “no opinion”. A sensitivity analysis excluding medical students led to minor changes in response patterns but did not substantially alter the overall perceptions of the program.

Previous research has also shown that healthcare providers perceive peer support as predominantly positive [27,28]. Yet despite recent advances in integrating such support into certified comprehensive care in some contexts, a lack of collaboration with self-help groups appears to persist [29]. Cited reasons for this are doubt in the effectiveness of peer support groups and concerns about bias or misinformation in this context [28], as well as limited resources [29] and a lack of visibility [28] or standardized processes regarding referrals to these groups [30]. In our study, both members and healthcare providers rated the negative effects of participation as low, as indicated by the TFA dimensions of burden and opportunity costs. However, we were able to confirm limited interaction between healthcare providers and PSGs, with only 5% of respondents ever having had contact with the Austrian Heart Association.

### Study Limitations

The following limitations need to be considered when interpreting this study’s findings:

Recruitment via institutional mailing lists, notice boards, and professional networks rather than personalized invitations prevented calculation of precise response rates and may have introduced selection bias. Additionally, unequal sample sizes between the PSG and control groups, as well as differences in baseline characteristics (particularly age, education level, and disease duration), pose a risk of confounding. These factors may influence perceived health literacy and reported quality of life independently of PSG participation. For example, older patients with longer disease histories may feel more confident in self-management due to accumulated experience, while educational differences may affect understanding and interpretation of health-related information.

Additionally, the sample size was based on the number of active members in the target population and realistic participation rates, rather than formal sample size calculation prior to the study. This may limit the statistical power and generalizability of the findings.

A further limitation is that the cardiovascular knowledge test had been recently developed. Although it underwent expert review with cognitive debriefing and was pilot-tested, it has not been subjected to full psychometric validation. Consequently, comparability with standardized international instruments is limited. Future research focused on validating and standardizing the instrument in larger and more-diverse populations would be beneficial in establishing its reliability and broader applicability.

Moreover, the reliance on self-reported data for certain variables may have introduced recall and social desirability biases.

Finally, the relatively small sample size and cross-sectional design limit causal inference and generalizability.

## 5. Conclusions

Cardiovascular PSGs that offer long-term CR programs provide valuable support, serving as an important social network. Participants in these groups feel more knowledgeable and empowered in managing their disease, and detrimental effects appear minimal. However, the lack of difference in objective CVD knowledge compared to non-members limits claims about enhanced health literacy. Further research is required to establish these effects, as well as the impact on personal health status, and to assess healthcare providers’ perceptions more extensively. However, referral to a PSG could be a valuable addition to the complex management of CVD patients, especially for those at risk of social isolation or depression. This could contribute to broader public health goals such as reducing the burden of CVD on healthcare systems by improving self-management.

## Figures and Tables

**Table 1:** Demographic and clinical characteristics of CVD patients.

Variable		PSG Members (*n* = 251)	Control Group (*n* = 76)	*p*-Value (Test Statistic)
Age (years, median (IQR)		74.00 (12)	59.00 (23)	<0.001 (U = 2263.00) *
Gender (%)	female	59.0	48.7	0.007 ^†^
	male	41.0	47.4	
	no answer	0.0	3.9	
Marital Status (%)	married/in a relationship	59.4	72.4	0.024 ^†^
	divorced/widowed	33.5	17.1	
	single	5.2	9.2	
	no answer	2.0	1.3	
Highest Level of Education (%)	primary school	6.8	0.0	<0.001 *^†^
	lower secondary education	12.7	9.2	
	apprenticeship/vocational school	50.6	44.7	
	high school	16.3	11.8	
	university degree	12.4	34.2	
	no answer	1.2	0.0	
Years Since Onset of CVD (%)	<5 years	8.4	14.5	0.008 (χ^2^(5) = 14.911)
	5–10 years	21.9	23.7	
	11–20 years	17.9	5.3	
	21–30 years	12.0	5.3	
	>30 years	5.2	2.6	
	no answer	34.7	48.7	

Note: Values represent percentages or medians ± IQR, as appropriate. Group sizes are shown in column headings. * Statistically significant difference (*p* < 0.05). ^†^ Fisher’s Exact Test was used where expected cell counts were <5; no test statistic is reported, as the test is based on exact probabilities rather than a calculated test statistic.

**Table 2:** Patients’ self-reported CVD knowledge.

Parameter	Statistic	PSG Members (*n* = 229)	Control Group (*n* = 76)	*p*-Value (Test Statistic)
Self-reported CVD knowledge score (0–10) **^‡^**	Median	7.00	6.00	0.014 (U = 7088.00) *
	IQR	3	3	
	Mean	6.70	6.07	
	SD	±1.84	±1.98	

**^‡^** Patients responding to the question “How would you rate your knowledge of cardiovascular diseases? (Please choose a number; higher numbers indicate better knowledge)”. * Statistically significant difference (*p* < 0.05).

**Table 3:** Respondents’ main sources of medical information on CVD and healthy lifestyle.

Parameter	Answer Categories	PSG Members (%, *n* = 251)	Control Group (%, *n* = 76)	*p*-Value (Test Statistic)
Main sources of medical information on CVD and healthy lifestyle **^‡^**	Austrian Heart Association	76.9	11.8	<0.001 (χ^2^(1) = 104.538) *
	Specialist	72.9	63.2	0.102 (χ^2^(1) = 2.674)
	General practitioner/family doctor	53.8	52.6	0.860 (χ^2^(1) = 0.031)
	Internet	17.5	32.9	0.004 (χ^2^(1) = 8.272) *
	Magazines	17.1	14.5	0.585 (χ^2^(1) = 0.299)
	Television	12.0	13.2	0.779 (χ^2^(1) = 0.079)
	Friends	6.0	18.4	<0.001 (χ^2^(1) = 11.179) *
	Acquaintances	2.8	6.6	0.159 ^†^
	Radio	1.2	2.6	0.330 ^†^
	Other	1.6	11.8	<0.001 *^†^

**^‡^** Patients responding to the question “Where do you get most of your medical information about your CVD or healthy lifestyle?”. * Statistically significant difference (*p* < 0.05). Participants could make up to three selections. ^†^ Fisher’s Exact Test was used where expected cell counts were <5; no test statistic is reported, as the test is based on exact probabilities rather than a calculated test statistic.

**Table 4:** Overview of participants’ self-reported health parameters.

Parameter	Category	PSG Members (%)	Control Group (%)	*p*-Value (Test Statistic)
BMI		*n* = 231	*n* = 64	0.525 ^†^
	Underweight (<18.5 kg/m^2^)	1.3	0.0	
	Normal weight (18.5–24.9 kg/m^2^)	35.5	39.1	
	Pre-obesity (25–29.9 kg/m^2^)	46.8	35.9	
	Class I obesity (30–34.9 kg/m^2^)	12.1	17.2	
	Class II obesity (35–39.9 kg/m^2^)	2.6	4.7	
	Class III obesity (≥40 kg/m^2^)	1.7	3.1	
Blood Pressure		*n* = 159	*n* = 44	0.037 (χ^2^(3) = 8.463)
	Normal (<130/85 mmHg)	43.4	36.4	
	Normal–high (130–139/85–89 mmHg)	28.3	29.5	
	Grade I hypertension (140–159/90–99 mmHg)	26.4	22.7	
	Grade II hypertension (≥160/100 mmHg)	1.9	11.4	
Blood Sugar Level		*n* = 79	*n* = 23	0.577 (χ^2^(1) = 0.312)
	Normal (HbA1c < 6.5% or FBS < 100 mg/dL)	45.6	52.2	
	Elevated (HbA1c ≥ 6.5% or FBS ≥ 100 mg/dL)	54.4	47.8	
Blood Lipid Level		*n* = 79	*n* = 17	0.758 (χ^2^(1) = 0.214)
	Normal (TC < 200 mg/dL, LDL-C < 160 mg/dL, or TG < 150 mg/dL)	75.9	70.6	
	Elevated (TC ≥ 200 mg/dL, LDL-C ≥ 160 mg/dL, or TG ≥ 150 mg/dL)	24.1	29.4	

BMI = body mass index; FBS = fasting blood sugar; HbA1c = hemoglobin A1c; TC = total cholesterol; LDL-C = low-density-lipoprotein cholesterol; TG = triglyceride. ^†^ Fisher’s Exact Test was used where expected cell counts were <5; no test statistic is reported, as the test is based on exact probabilities rather than a calculated test statistic.

## Data Availability

The datasets generated and analyzed during the current study are available from the corresponding author upon request.

## Abbreviations

The following abbreviations are used in this manuscript:

AHA	Austrian Heart Association (Österreichischer Herzverband)
CR	Cardiac rehabilitation
CVD	Cardiovascular disease
HRQoL	Health-related quality of life
PSG	Patient support group
TFA	Theoretical Framework of Acceptability

## Appendix A

### Appendix A.1. Participant Characteristics

**Table A1:** Distribution of healthcare providers by profession.

Healthcare Profession	Frequency (*n*)	Frequency (%)
General Practitioner	75	33.9
Registered Nurse	50	22.6
Occupational therapist	3	1.4
Surgical Specialist	1	0.5
Specialist for Internal Medicine	17	7.7
Physiotherapist	18	8.1
Medical student	27	12.2
Other	30	13.6
Total	221	100

### Appendix A.2. Health Literacy

**Table A2:** Patients’ perceived informedness about own illness.

Parameter	Answer Categories	PSG Members (%, *n* = 235)	Control Group (%, *n* = 60)	*p*-Value (Test Statistic)
Perceived informedness about own illness **^‡^**	I feel very well informed	40.0	26.7	<0.001 (χ^2^(3) = 23.339) *
	I feel well informed	53.2	46.7	
	I feel moderately informed	6.8	23.3	
	I don’t feel very well informed	0.0	3.3	
	I don’t feel well informed at all	0.0	0.0	

**^‡^** Patients responding to the question “How well informed do you feel about being diagnosed with your condition?”. * Statistically significant difference (*p* < 0.05).

**Table A3:** Patients’ knowledge about CVD risk factors and symptoms (% answering correctly).

Question Category	Correct Answer	PSG Members (%, *n* = 251)	Control Group (%, *n* = 76)	*p*-Value (Test Statistic)
Risk factors for CVD	Vitamin deficiency (“no”)	94.8	92.1	0.403 ^†^
	High blood pressure (“yes”)	94.0	86.8	0.039 (χ^2^(1) = 4.261) *
	Being overweight (“yes”)	85.7	88.2	0.579 (χ^2^(1) = 0.307)
	Smoking (“yes”)	84.5	92.1	0.090 (χ^2^(1) = 2.872)
	Lack of exercise (“yes”)	79.3	86.8	0.141 (χ^2^(1) = 2.169)
	High blood lipid levels (“yes”)	79.3	80.3	0.853 (χ^2^(1) = 0.034)
	Stress (“yes”)	72.9	76.3	0.554 (χ^2^(1) = 0.349)
	Diabetes (“yes”)	51.4	56.6	0.428 (χ^2^(1) = 0.629)
	Poor dietary habits (“yes”)	50.2	64.5	0.029 (χ^2^(1) = 4.778) *
	Sleep disorders (“yes”)	20.3	32.9	0.023 (χ^2^(1) = 5.172) *
Signs of a heart attack	Fever (“no”)	98.0	100.0	0.594 ^†^
	Tightness/pressure on the chest (“yes”)	82.5	86.8	0.368 (χ^2^(1) = 0.809)
	Chest pain (“yes”)	76.1	75.0	0.845 (χ^2^(1) = 0.038)
	Pain in the left arm (“yes”)	70.9	71.1	0.982 (χ^2^(1) = 0.001)
	Anxiety/feelings of apprehension/fear of dying (“yes”)	62.2	47.4	0.022 (χ^2^(1) = 5.259) *
	Shortness of breath (“yes”)	57.0	57.9	0.887 (χ^2^(1) = 0.020)
	Cold sweats (“yes”)	46.2	43.4	0.668 (χ^2^(1) = 0.184)
	Palpitations (“yes”)	44.2	44.7	0.937 (χ^2^(1) = 0.006)
	Nausea/vomiting (“yes”)	30.3	26.3	0.506 (χ^2^(1) = 0.442)
	Upper abdominal pain (“yes”)	21.5	28.9	0.179 (χ^2^(1) = 1.807)
	Pain in the neck/jaw area (“yes”)	20.7	13.2	0.141 (χ^2^(1) = 2.169)
	Dizziness (“yes”)	15.9	30.3	0.006 (χ^2^(1) = 7.698) *
Signs of a stroke	Drooping mouth corner (“yes”)	86.9	86.8	0.998 (χ^2^(1) = 0.000)
	Speech disorder (“yes”)	84.5	92.1	0.090 (χ^2^(1) = 2.872)
	One-sided arm paralysis (“yes”)	72.5	76.3	0.511 (χ^2^(1) = 0.433)
	One-sided facial paralysis (“yes”)	70.1	75.0	0.410 (χ^2^(1) = 0.678)
	One-sided sensory disturbance (“yes”)	57.8	65.8	0.212 (χ^2^(1) = 1.559)
	Sudden one-sided vision disturbances (“yes”)	49.8	48.7	0.865 (χ^2^(1) = 0.029)
	Balance disorder (“yes”)	39.4	48.7	0.152 (χ^2^(1) = 2.051)
	Sudden severe headache (“yes”)	34.7	38.2	0.577 (χ^2^(1) = 0.312)
	Dizziness (“yes”)	25.9	32.9	0.231 (χ^2^(1) = 1.432)

^†^ Fisher’s Exact Test was used where expected cell counts were <5; no test statistic is reported, as the test is based on exact probabilities rather than a calculated test statistic. * Statistically significant difference (*p* < 0.05).

### Appendix A.3. Self-Reported Health Data

**Table A4:** Medical conditions reported by participants.

Parameter	Answer Categories	PSG Members (%, *n* = 249)	Control Group (%, *n* = 70)	*p*-Value (Test Statistic)
	High blood pressure	52.2	47.1	0.454 (χ^2^(1) = 0.561)
Self-reported medical conditions **^‡^**	Heart failure	38.2	15.7	<0.001 (χ^2^(1) = 12.399)
	Coronary artery disease	35.3	15.7	0.002 (χ^2^(1) = 9.834)
	Overweight	28.5	27.1	0.822 (χ^2^(1) = 0.051)
	Osteoarthritis	21.3	12.9	0.115 (χ^2^(1) = 2.479)
	Arteriosclerosis	18.9	11.4	0.145 (χ^2^(1) = 2.124)
	Diabetes	16.1	17.1	0.829 (χ^2^(1) = 0.047)
	Peripheral arterial disease	14.9	11.4	0.466 (χ^2^(1) = 0.531)
	Other heart diseases	11.2	8.6	0.522 (χ^2^(1) = 0.410)
	Cancer	10.4	2.9	0.048 (χ^2^(1) = 3.926)
	Rheumatism	10.4	10.0	0.915 (χ^2^(1) = 0.011)
	Kidney disease	6.0	1.4	0.210 ^†^
	COPD	5.2	2.9	0.536 ^†^
	Depression	3.2	15.7	<0.001 ^†^
	Parkinson’s disease	2.0	1.4	1 ^†^
	Dementia	1.6	1.4	1 ^†^

**^‡^** Patients responding to the question “What medical conditions are you known to have?”. Participants could make multiple selections. ^†^ Fisher’s Exact Test was used where expected cell counts were <5; no test statistic is reported, as the test is based on exact probabilities rather than a calculated test statistic.

**Table A5:** Number of medical conditions reported by participants.

Parameter	Categories	PSG Members (%, *n* = 249)	Control Group (%, *n* = 70)	*p*-Value (Test Statistic)
Number of self-reported medical conditions	0–2	48.2	68.6	0.015 ^†^
	3–6	49.0	30.0	
	7–10	2.0	1.4	
	>10	0.8	0.0	

Percentage of participants in each category based on their responses to the question “Which medical conditions have you been diagnosed with?”. Participants could make multiple selections. ^†^ Fisher’s Exact Test was used where expected cell counts were <5; no test statistic is reported, as the test is based on exact probabilities rather than a calculated test statistic.

**Table A6:** Self-reported duration of cardiovascular disease.

Parameter	Statistic	PSG Members (*n* = 168)	Control Group (*n* = 42)	*p*-Value (Test Statistic)
Duration of cardiovascular disease (years)	Median	12.0	5.0	0.001 (U = 2264.00)
	IQR	15	7	
	Mean	15.05	9.39	
	SD	11.65	9.74	

Duration of cardiovascular disease among participants based on their responses to the question “How long have you had a cardiovascular disease?”.

**Table A7:** Self-reported number of medications taken by participants.

Parameter	Statistic	PSG Members (%, *n* = 163)	Control Group (%, *n* = 44)	*p*-Value (Test Statistic)
Number of medications taken regularly	Median	3.00	2.00	*p* = 0.008 (U = 2663.50)
	IQR	3.0	3.8	
	Mean	3.70	2.80	
	SD	2.29	2.26	

Percentage of participants in each category based on their responses to the question “Which medications do you take regularly?”.

**Table A8:** Disclosure of personal health data by participants.

Parameter		PSG Members (%, *n* = 251)	Control Group (%, *n* = 76)	*p*-Value (Test Statistic)
BMI (weight and height provided)		92.0	84.2	0.044 (χ^2^(1) = 4.042)
Blood Pressure (systolic and diastolic values provided)		57.4	57.9	0.935 (χ^2^(1) = 4.042)
Blood Sugar Levels	FBS	29.9	28.9	0.876 (χ^2^(1) = 0.024)
	HbA1c	21.1	17.1	0.445 (χ^2^(1) = 0.582)
Blood Lipid Levels	TC	30.7	21.1	0.103 (χ^2^(1) = 2.655)
	LDL-C	28.3	18.4	0.086 (χ^2^(1) = 2.952)
	TG	27.1	21.1	0.291 (χ^2^(1) = 1.114)

Percentage of participants providing data on their personal health data. BMI = body mass index; HbA1c = hemoglobin A1c; FBS = fasting blood sugar; TC = total cholesterol; LDL-C = low-density-lipoprotein cholesterol; TG = triglyceride.

### Appendix A.4. Health-Related Quality of Life

**Table A9:** Health-related Quality of Life (EQ-5D-5L index).

Parameter	Statistic	PSG Members (*n* = 226)	Control Group (*n* = 64)	*p*-Value (Test Statistic)
Health-related Quality of Life (EQ-5D-5L index)	Median	0.943	0.913	0.251 (U = 6562.00)
	IQR	0.120	0.173	
	Mean	0.912	0.875	
	SD	0.101	0.153	

**Table A10:** Participants’ perception of their Health-Related Quality of Life (HRQoL).

Domain	Response Options	PSG Members (%)	Control Group (%)	*p*-Value (Test Statistic)
Mobility		*n* = 241	*n* = 65	
	I have no problems walking around	75.5	69.2	0.130 ^†^
	I have slight problems walking around	17.8	16.9	
	I have moderate problems walking around	5.8	9.2	
	I have severe problems walking around	0.8	4.6	
	I am unable to walk around	0.0	0.0	
Independence		*n* = 240	*n* = 67	
	I have no problems washing myself or getting dressed	93.8	94.0	0.907 ^†^
	I have slight problems washing myself or getting dressed	4.6	6.0	
	I have moderate problems washing myself or getting dressed	1.3	0.0	
	I have great difficulty washing or dressing myself	0.4	0.0	
	I am unable to wash or dress myself	0.0	0.0	
Daily Activities		*n* = 240	*n* = 67	
	I have no problems performing my daily activities	82.1	80.6	0.316 ^†^
	I have slight problems performing my daily activities	14.2	11.9	
	I have moderate problems performing my daily activities	2.9	4.5	
	I have great problems performing my daily activities	0.0	1.5	
	I am unable to perform my daily activities	0.8	1.5	
Pain/Physical discomfort		*n* = 233	*n* = 67	
	I have no pain or discomfort.	36.9	43.3	0.512 (χ^2^(3) = 2.303)
	I have mild pain or discomfort.	39.9	29.9	
	I have moderate pain or discomfort.	21.0	23.9	
	I have severe pain or discomfort.	2.1	3.0	
	I have extreme pain or discomfort.	0.0	0	
Anxiety/Depression		*n* = 235	*n* = 66	
	I am not anxious or depressed.	68.9	48.5	<0.001 (χ^2^(3) = 18.077)
	I am a little anxious or depressed.	24.3	30.3	
	I am moderately anxious or depressed.	6.0	13.6	
	I am very anxious or depressed.	0.9	7.6	
	I am extremely anxious or depressed.	0.0	0.0	

^†^ Fisher’s Exact Test was used where expected cell counts were <5; no test statistic is reported, as the test is based on exact probabilities rather than a calculated test statistic.

**Table A11:** Self-rated current health status among PSG members and control group.

Parameter	Statistic	PSG Members (*n* = 235)	Control Group (*n* = 63)	*p*-Value (Test Statistic)
EQ-5D Visual Analog Scale Health Score (0–100)	Median	80.00	75.00	0.484 (U = 6982.50)
	IQR	25	38	
	Mean	72.6	70.5	
	SD	±17.7	±19.7	

### Appendix A.5. Perception of the Program

**Table A12:** PSG members’ attitudes towards the AHA as assessed using the TFA.

Domain	Response Options	*n* (Responses)	% of Respondents
Affective attitude I *“Do you like or dislike the program of the AHA?”*	Strongly dislike	1	0.6
	Dislike	2	1.3
	No opinion	5	3.2
	Like	59	38.1
	Strongly like	88	56.8
	Total	155	100
Affective attitude II *“How comfortable do you feel participating in the activities of the AHA?”*	Very uncomfortable	7	3.2
	Uncomfortable	1	0.5
	No opinion	8	3.6
	Comfortable	79	35.9
	Very comfortable	125	56.8
	Total	220	100
Burden *“How much effort does it take you to participate in the activities of the AHA?”*	No effort at all	102	45.1
	A little effort	97	42.9
	No opinion	12	5.3
	A lot of effort	13	5.8
	Huge effort	2	0.9
	Total	226	100
Ethicality I *“There are moral or ethical consequences to participating in the activities of the AHA.”*	Strongly disagree	39	19.0
	Disagree	16	7.8
	No opinion	36	17.6
	Agree	69	33.7
	Strongly agree	45	22.0
	Total	205	100
Ethicality II *“How fair is the program of the AHA for people with cardiovascular diseases?”*	Very unfair	4	1.8
	Unfair	0	0.0
	No opinion	13	5.9
	Fair	93	41.9
	Very Fair	112	50.5
	Total	222	100
Perceived effectiveness *“Participating in the activities of the AHA has strengthened me in managing my cardiovascular disease.”*	Strongly disagree	0	0.0
	Disagree	3	1.4
	No opinion	28	13.1
	Agree	86	40.4
	Strongly agree	96	45.1
	Total	213	100
Intervention coherence *“It is clear to me how participating in the activities of the AHA will help me manage my cardiovascular disease.”*	Strongly disagree	1	0.5
	Disagree	5	2.3
	No opinion	23	10.7
	Agree	111	51.9
	Strongly agree	74	34.6
	Total	214	100
Self-efficacy *“How confident do you feel about your ability to participate in the activities of the AHA?”*	Very unconfident	5	2.2
	Unconfident	12	5.4
	No opinion	19	8.5
	Confident	108	48.4
	Very confident	79	35.4
	Total	223	100
Opportunity costs *“Participating in the activities of the AHA interferes with my other priorities.”*	Strongly disagree	99	45.4
	Disagree	76	34.9
	No opinion	26	11.9
	Agree	9	4.1
	Strongly agree	8	3.7
	Total	218	100
General Acceptability *“How acceptable is the program of the AHA to you?”*	Completely unacceptable	8	3.6
	Unacceptable	1	0.5
	No opinion	11	5.0
	Acceptable	75	33.9
	Completely acceptable	126	57.0
	Total	221	100

**Table A13:** PSG members’ perceptions of the benefits of participating in the AHA.

Statement	Responses Options	*n* (Responses)	% of Respondents
Participating in the activities of the Heart Association strengthens me in my ability to deal with my own illness.	Yes	129	52.2
	No	118	47.8
	Total	247	100
The Heart Association constitutes an important social network for me and gives me strength in living with the illness.	Yes	129	52.2
	No	118	47.8
	Total	247	100
The Heart Association provides me with support that I do not receive from any doctor or other healthcare provider.	Yes	56	22.7
	No	191	77.3
	Total	247	100
The Heart Association is an important source of trustworthy medical information.	Yes	119	48.2
	No	128	51.8
	Total	247	100
Membership in the Heart Association helps me navigate the healthcare system.	Yes	80	32.4
	No	167	67.6
	Total	247	100
Participating in the activities of the Heart Association improves my quality of life.	Yes	172	69.6
	No	75	30.4
	Total	247	100

Participants were asked to tick statements they agreed with. “Yes” indicates the statement was selected; “No” indicates it was not selected.

**Table A14:** Healthcare providers’ attitudes towards the AHA as assessed using the TFA.

Domain	Response Options	*n* (Responses)	% of Respondents
Affective attitude *“Do you like or dislike the activities of the AHA?”*	Strongly dislike	2	1.6
	Dislike	3	2.4
	No opinion	82	66.7
	Like	30	24.4
	Strongly like	6	4.9
	Total	123	100
Burden *“How much effort does it take you to collaborate with the AHA?”*	No effort at all	46	40.4
	A little effort	21	18.4
	No opinion	45	39.5
	A lot of effort	2	1.8
	Huge effort	0	0.0
	Total	114	100
Ethicality *“There are moral or ethical consequences to participating in the activities of the AHA.”*	Strongly disagree	24	19.5
	Disagree	30	24.4
	No opinion	67	54.5
	Agree	1	0.8
	Strongly agree	1	0.8
	Total	123	100
Perceived effectiveness *“Participating in the activities of the AHA has strengthened its members in managing their cardiovascular disease.”*	Strongly disagree	0	0.0
	Disagree	8	6.3
	No opinion	58	45.7
	Agree	48	37.8
	Strongly agree	13	10.2
	Total	127	100
Intervention coherence *“It is clear to me how participating in the activities of the AHA will help patients with cardiovascular disease.”*	Strongly disagree	8	5.4
	Disagree	16	10.9
	No opinion	50	34.0
	Agree	59	40.1
	Strongly agree	14	9.5
	Total	147	100
Self-efficacy *“How confident do you feel about patients’ ability to participate in the activities of the AHA?”*	Very unconfident	5	3.5
	Unconfident	18	12.5
	No opinion	48	33.3
	Confident	62	43.1
	Very confident	11	7.6
	Total	144	100
Opportunity costs *“Collaborating with the AHA interferes with my other priorities.”*	Strongly disagree	27	22.1
	Disagree	33	27.0
	No opinion	57	46.7
	Agree	4	3.3
	Strongly agree	1	0.8
	Total	122	100
General Acceptability *“How acceptable is the program of the AHA to you?”*	completely unacceptable	0	0.0
	unacceptable	0	0.0
	no opinion	67	55.8
	acceptable	40	33.3
	completely acceptable	13	10.8
	Total	120	100

## Appendix B

### Sensitivity Analysis

**Table A15:** Healthcare providers’ attitudes towards the AHA as assessed using the TFA–sensitivity analysis excluding medical students (*n* = 27).

Domain	Response Options	*n* (Responses)	% of Respondents	Δ % *
Affective attitude *“Do you like or dislike the activities of the AHA?”*	Strongly dislike	1	0.9	−0.7
	Dislike	3	2.8	+0.4
	No opinion	73	67.6	+0.9
	Like	26	24.1	−0.3
	Strongly like	5	4.6	−0.3
	Total	108	100	n/a
Burden *“How much effort does it take you to collaborate with the AHA?”*	No effort at all	41	41.0	+0.6
	A little effort	19	19.0	+0.6
	No opinion	38	38.0	−1.5
	A lot of effort	2	2.0	+0.2
	Huge effort	0	0.0	0
	Total	100	100	n/a
Ethicality *“There are moral or ethical consequences to participating in the activities of the AHA.”*	Strongly disagree	23	21.3	+1.8
	Disagree	24	22.2	−2.2
	No opinion	60	55.6	+1.1
	Agree	1	0.9	+0.1
	Strongly agree	0	0.0	+0.8
	Total	108	100	n/a
Perceived effectiveness *“Participating in the activities of the AHA has strengthened its members in managing their cardiovascular disease.”*	Strongly disagree	0	0.0	0
	Disagree	7	6.2	−0.1
	No opinion	52	46.0	+0.3
	Agree	42	37.2	−0.6
	Strongly agree	12	10.6	+0.4
	Total	113	100	n/a
Intervention coherence *“It is clear to me how participating in the activitites of the AHA will help patients with cardiovascular disease.”*	Strongly disagree	6	4.7	−0.7
	Disagree	13	10.1	−0.7
	No opinion	44	34.4	+0.4
	Agree	52	39.8	−0.3
	Strongly agree	14	10.9	+1.4
	Total	129	100	n/a
Self-efficacy *“How confident do you feel about patients’ ability to participate in the activities of the AHA?”*	Very unconfident	4	3.1	−0.4
	Unconfident	15	11.8	−0.7
	No opinion	43	33.9	+0.6
	Confident	57	44.1	+1.0
	Very confident	9	7.1	−0.5
	Total	128	100	n/a
Opportunity costs *“Collaborating with the AHA interferes with my other priorities.”*	Strongly disagree	27	25.0	+2.9
	Disagree	28	25.9	−1.1
	No opinion	49	45.4	−1.3
	Agree	3	2.8	−0.5
	Strongly agree	1	0.9	+0.1
	Total	108	100	n/a
General Acceptability *“How acceptable is the program of the AHA to you?”*	completely unacceptable	0	0.0	0
	unacceptable	0	0.0	0
	no opinion	58	55.8	0
	acceptable	35	32.7	−0.6
	completely acceptable	12	11.5	+0.7
	Total	105	100	n/a

* Difference in percentage in relation to the whole population of healthcare providers (including medical students).
